# Supplementary material for: Modeling glioblastoma heterogeneity as a dynamic network of cell states
Source: Mol Syst Biol. 2021 Sep 16;17(9):e10105. doi: 10.15252/msb.202010105 (PMC8444284; doi:10.15252/msb.202010105)
Supplement: Supplementary file 5 — Source Data for Figure 3 [file MSB-17-e10105-s001.zip › Figure3A_sourcedata/GSEA_3065/hallmarks_state4.GseaPreranked.1623416296068/HALLMARK_KRAS_SIGNALING_DN.html]

Details for gene set HALLMARK\_KRAS\_SIGNALING\_DN[GSEA]

|  || Dataset | state4 |
| Phenotype | NoPhenotypeAvailable |
| Upregulated in class | na\_pos |
| GeneSet | HALLMARK\_KRAS\_SIGNALING\_DN |
| Enrichment Score (ES) | 0.42869616 |
| Normalized Enrichment Score (NES) | 1.3050575 |
| Nominal p-value | 0.13031915 |
| FDR q-value | 0.21007387 |
| FWER p-Value | 0.689 |
Table: GSEA Results Summary

  

Fig 1: Enrichment plot: HALLMARK\_KRAS\_SIGNALING\_DN      
 Profile of the Running ES Score & Positions of GeneSet Members on the Rank Ordered List

  

| PROBE | GENE SYMBOL | GENE\_TITLE | RANK IN GENE LIST | RANK METRIC SCORE | RUNNING ES | CORE ENRICHMENT || 1 | KLHDC8A |  |  | 300 | 0.174 | 0.0462 | Yes |
| 2 | SLC25A23 |  |  | 361 | 0.162 | 0.1245 | Yes |
| 3 | SNN |  |  | 407 | 0.151 | 0.1990 | Yes |
| 4 | TGFB2 |  |  | 537 | 0.132 | 0.2501 | Yes |
| 5 | CCDC106 |  |  | 591 | 0.127 | 0.3102 | Yes |
| 6 | BTG2 |  |  | 689 | 0.116 | 0.3574 | Yes |
| 7 | EFHD1 |  |  | 869 | 0.098 | 0.3819 | Yes |
| 8 | GAMT |  |  | 971 | 0.090 | 0.4145 | Yes |
| 9 | PDK2 |  |  | 1354 | 0.068 | 0.3904 | Yes |
| 10 | NUDT11 |  |  | 1509 | 0.061 | 0.3990 | Yes |
| 11 | COPZ2 |  |  | 1530 | 0.061 | 0.4287 | Yes |
| 12 | KCNQ2 |  |  | 2023 | 0.046 | 0.3751 | No |
| 13 | SLC38A3 |  |  | 2359 | 0.038 | 0.3420 | No |
| 14 | SKIL |  |  | 2374 | 0.038 | 0.3601 | No |
| 15 | BMPR1B |  |  | 2711 | 0.030 | 0.3227 | No |
| 16 | FGFR3 |  |  | 2779 | 0.029 | 0.3277 | No |
| 17 | LFNG |  |  | 3245 | 0.020 | 0.2640 | No |
| 18 | TCF7L1 |  |  | 3268 | 0.019 | 0.2709 | No |
| 19 | PTPRJ |  |  | 3822 | -0.000 | 0.1823 | No |
| 20 | CELSR2 |  |  | 3946 | -0.007 | 0.1661 | No |
| 21 | IDUA |  |  | 4172 | -0.020 | 0.1409 | No |
| 22 | IGFBP2 |  |  | 5777 | -0.179 | -0.0193 | No |
| 23 | BARD1 |  |  | 5779 | -0.179 | 0.0776 | No |
Table: GSEA details [plain text format]

  

Fig 2: HALLMARK\_KRAS\_SIGNALING\_DN: Random ES distribution      
 Gene set null distribution of ES for **HALLMARK\_KRAS\_SIGNALING\_DN**
